# Testing the concurrent validity and reliability of a lipowise digital skinfold caliper to assess muscle mass in healthy young adults

**DOI:** 10.1016/j.heliyon.2023.e17569

**Published:** 2023-06-22

**Authors:** César Leão, Filipe Manuel Clemente, Bruno Silva, Joel Pereira, Georgian Badicu, Miguel Camões, José Maria Cancela

**Affiliations:** a University of Vigo, Faculty of Educational Sciences and Sports Sciences, 36005 Pontevedra, Spain; b Escola Superior Desporto e Lazer, Instituto Politécnico de Viana do Castelo, Rua Escola Industrial e Comercial de Nun’Álvares, 4900-347 Viana do Castelo, Portugal; c Research Center in Sports Performance, Recreation, Innovation and Technology (SPRINT), 4960-320 Melgaço, Portugal; d Instituto de Telecomunicações, Delegação da Covilhã, Lisboa 1049-001, Portugal; e Department of Physical Education and Special Motricity, Faculty of Physical Education and Mountain Sports, Transilvania University of Braşov, 500068 Braşov, Romania

**Keywords:** Body composition, Anthropometry, Muscle mass, Skinfold calipers

## Abstract

The aim of this study was to assess the validity and reliability of a novel tool to assess skinfolds and to compare the muscle mass measured through dual-x-ray-absorptiometry (DXA) and estimated using the Lee equation from the values of the skinfolds and girths in a healthy young adult population. Methods: The present study followed a cross-sectional design, including 38 participants, with 27 males (22.04 ± 5.20 years) and 11 females (21.55 ± 2.39 years). The measurement protocol included a DXA evaluation, basic measurements of body mass and stature, eight skinfolds with two skinfold calipers of different brands (Harpenden and Lipowise), and three girths. The order in which the skinfold calipers were used was randomized. The muscle mass was then calculated using the formula established by Lee et al. Results: No significant differences were found between the two skinfold calipers considering all the outcomes (p > 0.05). The correlation coefficients were between 0.724 and 0.991, which suggest very-large to nearly perfect correlations. The correlations performed revealed that muscle mass estimated from DXA is nearly perfectly correlated with both muscle mass estimated from the data obtained with the Harpenden skinfold caliper (r = 0.955) and muscle mass estimated from the data obtained with the Lipowise skinfold caliper (r = 0.954). From the results, we conclude that Lipowise caliper is an accurate skinfold caliper and it can be an alternative tool for the technician that need to assess body fat or muscle mass in precise, valid and time efficient evaluation. It should be noted that the caution to use skinfold calipers interchangeable with each other when evaluating skinfolds remains a necessity and is advisable to perform the measurements with the same brand and model of skinfold caliper when the purpose is to perform follow-up assessments.

## 1 Introduction

Body composition can be approached based on five levels: atomic, molecular, cellular, tissue, and whole body. Because of this organization, there are implications in choosing a method for the intended evaluation [1]. As in clinical and research contexts, molecular and tissue models are usually the primary models used to assess body composition [2,3]. This categorization also leads to a compartmentalization of body tissues in the description of body composition, causing different models to have different numbers of components [4]. Of the various components that can be evaluated, the percentage of fat mass and the amount of muscle mass are thought to be the most important aspects in assessing the risk of injury and health risks [5]. Whether their ratio has an optimal relationship with athletes’ performance is also an important consideration [6].

It is commonly accepted that body composition assessment methods are classified into reference methods, laboratory methods, and field methods [7]. However, several factors must be considered when choosing a body composition assessment method. These include not only technical issues, such as validity, safety, accuracy, and reliability assessments, but also more practical factors, such as availability, portability, financial implications, time availability, invasion of privacy, and technical knowledge to conduct the method [8,9].

Currently, the most accepted method, despite its limitations, for evaluating bone mass, fat mass, and lean body mass in healthy adults is dual-energy X-ray absorptiometry (DXA) [9], but it is considered too expensive for most situations [7]. A more practical, although less precise, way to evaluate body composition is anthropometry. Measurements of some human body dimensions that use surface landmarks as a reference allow values to be obtained that can be converted to body fat percentage or muscle mass using equations [3]. Rigorous uniformization protocols are required to guarantee the reliability of the values found in such evaluations [2]. Many protocols have been proposed, such as that developed by Marfell-Jones, which is currently the most-used protocol by investigators [10], with the goals of limiting the variation in measurements and minimizing technical errors in measurements.

Of all the items needed to ensure the validity of the protocol, it is necessary to use an instrument that can ensure constant pressure throughout the evaluation [11]. Among available skinfold calipers, the Harpenden skinfold caliper is considered by most anthropometrists as the best criterion instrument and is the most popular in the scientific field [12,13].

Harpenden was one of the first skinfold calipers to be created and is one of the most-quoted devices in scientific papers [12]. It features a measurement readout dial with a resolution of 0.2 mm and a measurement range of 80 mm despite being initially designed with an aperture of 40 mm. Its body is made of stainless steel covered with polymeric parts. Its manufacturers report that it has a compression of 10 g/mm^2^ in new calipers. Furthermore, the Harpenden caliper exhibited the best correlation against other calipers when the assessment followed the protocol and was performed by an experienced anthropometrist [14].

Lipowise is a patented digital skinfold caliper classified as a medical device that is connectable with iOS/Android APPs, that provides several features, including 48 equations applicable to 15 possible skinfolds to estimate body fat percentage. Lipowise applies a constant compression force of 10 gf/mm^2^ (error<5%) with a resolution of 0.1 mm (error<5%) at a sample rate of 100 Hz (100 values/second), which allows the profile to be traced and the tissue compressibility to be analyzed [15,16]. The PRO version has an aluminum body and a measurement range of 100 mm; the LIGHT version has a polymeric body with a measurement range of 50 mm.

Despite the potential benefits of Lipowise, only a few reports of concurrent validity and reliability have been reported so far [17]. Amaral and coworkers tested the beta version of the LipoTool before the commercial version of Lipowise was released. This could be an opportunity for developing research to assess the validity of the data extracted from the device. Research on concurrent validity and reliability will increase sports scientists' and practitioners’ confidence in the data obtained from the device. Thus, the aim of this study was two-fold: (i) to evaluate a novel skinfold caliper to assess skinfolds in order to assess its validity and reliability and (ii) to compare the body fat, fat-free mass and muscle mass measured through DXA and estimated by equations from the values of the skinfolds and girths.

## 2 Material and methods

This cross-sectional study was approved by the ethics committee in the School of Sport and Leisure—Viana do Castelo Polytechnic Institute with the code **CTC-ESDL-CE008**–**2021**. All participants were informed about the research protocol, requisites, benefits, and risks; their written consent was obtained before the beginning of the study. The study was conducted according to the Declaration of Helsinki (revised version of 2013 at the 64th WMA General Assembly, Fortaleza, Brazil) [18].

### 2.1 Participants

Convenience sampling was used as a sampling strategy. Thirty-eight university students were recruited (Table 1). The inclusion criteria were that participants (i) were physically active, (ii) did not have any condition that could change the anthropometric evaluations from the ISAK norms, and (iii) did not report any drug consumption or hormonal or corticosteroid treatment. The exclusion criteria were that (i) performed the repeated measures with no missing dataand (ii) female participants were not within the second and third week of the menstrual cycle.

**Table 1:** Demographic information of the participants.

	Total (n = 38)	Female (n = 11)	Male (n = 27)
	Mean (SD)	Mean (SD)	Mean (SD)
Age (years)	21.9 (4.6)	21.6 (2.4)	22.0 (5.2)
Stature (cm)	171.9 (10.5)	161.2 (6.9)	176.3 (8.4)
Body mass (kg)	66.1 (11.2)	56.4 (9.1)	70.1 (9.5)
%MG DXA (%)	22.0 (7.3)	31.4 (3.7)	18.1 (4.2)
%BF Harp (%)	17.2 (7.6)	27.3 (3.6)	13.0 (4.0)
%BF Lipo (%)	16.8 (7.6)	27.2 (3.7)	12.6 (3.7)
FFM DXA (kg)	50.2 (10.5)	37.3 (4.7)	55.4 (7.1)
FFM Harp (kg)	55.1 (12.3)	40.8 (6.3)	61 (8.9)
FFM Lipo (kg)	55.4 (12.2)	40.9 (6.2)	61.3 (8.6)
Soma Har (mm)	84.1 (31.7)	117.4 (22.5)	70.3 (23.5)
Soma Lipo (mm)	86.0 (32.2)	120.0 (24.1)	72.2 (23.9)
MMLee harp (kg)	28.4 (6.2)	20.8 (2.7)	31.5 (4.3)
MMLee Lipo (kg)	28.4 (6.2)	20.7 (2.6)	31.5 (4.2)
MMDXA (kg)	27.1 (6.7)	18.8 (2.6)	30.4 (4.6)

%BF – body fat percentage; %BF Harp – body fat percentage estimated from values of Harpenden; %BF Lipo – body fat percentage estimated from values of Lipowise; FFM DXA – fat-free mass from DXA; FFM Harp – fat-free mass estimated from values of Harpenden; FFM Lipo – fat-free mass estimated from values of Lipowise; Soma Harp – skinfold sum of Harpenden values; Soma Lipo - skinfold sum of Lipowise values; MMLee Harp – muscle mass estimate with Lee equation from Harpenden values; MMLee Lipo - muscle mass estimate with Lee equation from Lipowise values; MMDXA – muscle mass estimate with ALST values from DXA.

### 2.2 Procedures and context

This study was conducted from October to December 2022. Participants were scheduled regarding the availability of the laboratory and whether they met the inclusion criteria. The room temperature was always 21 °C, and all evaluations were conducted in the morning between 9:30 a.m. and 12:30 p.m. to ensure that all conditions were the same.

Participants were instructed to arrive at the laboratory in a fasted state, abstain from coffee, refrain from practicing intense physical exercise in the previous 24 h, and wear light sports clothing (men: shorts; women: shorts and a sports top).

### 2.3 Stature and body mass assessment

Upon their arrival, each participant had their height measured to the nearest 0.1 cm with a portable stadiometer (Seca 217, Hamburg, Germany) and their weight measured to the nearest 0.1 kg with a mechanical floor scale (Seca 760, Hamburg, Germany). The measurements were obtained while participants were barefoot. Participants were measured by a single certified expert (ISAK Level 2), with a mean technical error of measurement (TEM) for these measurements of 0.01% [19].

### 2.4 DXA assessment

The participants were evaluated by a certified and experienced DXA operator using the DXA clinical method with a General Electric Hologic Discovery scanner (Hologic Inc., Waltham, MA, USA, Software version: QDR System Software version 12.4.2.). Prior to each scanning day, quality control procedures were realized as stated by the manufacturer's specification. In addition, participants presented themselves to the laboratory after a night without food intake in a rested state and with an empty bladder. All participants were instructed not to change their normal food intake on the previous day. Participants assumed a supine, stationary position on the equipment bed with both arms pronated by their sides and their head in the Frankfort plane, without using positioning aids. The DXA operator manually helped the participants [1] straighten their heads [2]; correct the position of their shoulders, pelvis, and legs [3]; place both arms in protonation by their sides; and [4] fix their feet together using strapping [20]. The main outcomes extracted from the DXA were the body fat percentage, fat-free mass, lean mass, and bone mass. The regional composition analysis of appendicular measures was done automatically by the software and confirmed by the technician. We used the equation by Kim et al. [21] to estimate muscle mass from the values obtained with DXA.

### 2.5 Skinfold assessment

After the DXA evaluation, the participants were measured by a single certified expert (ISAK Level 2), with a mean TEM for the skinfolds of 2.38% and 3.24% (for Harpenden and Lipowise skinfold calipers, respectively) [19], according to the protocol of the International Society for the Advancement of Kinanthropometry [10].

**Fig. 1:**
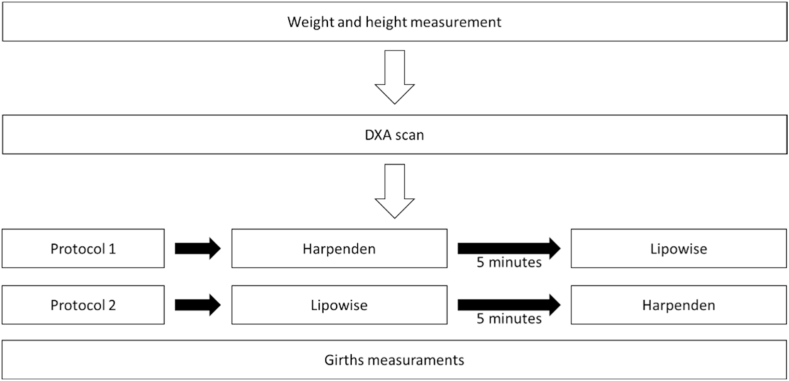
Assessments procedures.

The expert started the evaluation by marking the anthropometric landmarks and then measuring the skinfolds. Eight skinfolds (triceps, subscapular, biceps, suprailiac, abdominal, supraspinal, thigh, and calf) were measured to the nearest 0.1 mm with a Harpenden skinfold caliper (British Indicators, Ltd., London, UK) and a Lipowise skinfold caliper (Wisify, Porto, Portugal). The order of assessments using different equipment was organized so that they were used alternately; 5 min elapsed between evaluations with the different skinfold calipers (Fig. 1).

All skinfolds were evaluated three times, regardless of the difference between the first two evaluations; the final value was the median of the three measurements. The eight skinfolds were evaluated completely, followed by a second complete evaluation and ending with a third evaluation. The values were measured and stated aloud so the observer could record them. Even though the values in the Lipowise skinfold caliper are registered automatically, the final values of each evaluation were stated so the observer could register them.

Each set of skinfold measurements was taken sequentially in the order established by ISAK [10], and the reading was performed 2 s after the full pressure of the skinfold caliper was applied. The 2 s were counted by the evaluator. However, this was not necessary for the readings made with the Lipowise skinfold caliper because it uses software that can be programmed to read the value after 2 s.

After that, and with the set of skinfold values obtained from both pieces of equipment, the body fat percentage (%BF) was estimated using the equation developed by Eston et al. The value obtained for %BF was used to calculate fat-free mass (FFM).

Eston et al. equation for men = 1.61+(0.12*(Σ4skf)) + (0.36*(Σ2skf))

Eston et al. equation for women = 7.38+(0.07*(Σ4skf)) + (0.38*(Σ2skf))

Σ4skf – tricipital skf + subscapular skf + suprailiac skf + abdominal skf

Σ2skf – thigh skinfold + calf skinfold

**Fig. 2:**
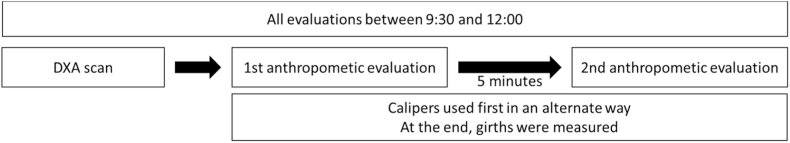
Schematic of the evaluations.

Finally, three girths were measured to the nearest mm with a Lufkin tape (Apex Tool Group, United States) to apply Lee et al.‘s equation [22] to assess muscle mass. The three girths (arm relaxed, thigh, and calf) were measured twice (Fig. 2), with the mean value used in the equation. The mean TEM for these variables was 0.07%.

Lee et al.‘s equation – Height (m)*(0.00744*CAG^2^ + 0.00088*CTG^2^ + 0.00441*CCG^2^) + 2.4*Sex – 0,048*Age (years) + Race + 7.8.

CAG – corrected arm girth; CTG – corrected thigh girth; CCG – corrected calf girth; Sex – 1 for male, 0 for female; Race – 0 for Caucasian, 1.1 for African American, 2 for Asian.

### 2.6 Statistical procedures

Descriptive statistics are presented as means and standard deviations. The within-instrument variability (considering the three trials performed) was tested using the coefficient of variation (%), the intraclass correlation (ICC) test using two-way random, absolute agreement, and the outcome in average measures and by executing a repeated-measures ANOVA after the normality (p > 0.05) and homogeneity (p > 0.05) of the sample were confirmed using the Shapiro-Wilk and Leven's tests, respectively.

Concurrent validity between instruments was evaluated using visual inspection through a Bland-Altman plot, supplemented by lower and upper limits established for a 95% confidence interval. Differences were also tested using repeated measures ANOVA, and the relationships were analyzed using the Pearson product-moment correlation. The following correlation scale was adopted [23]: trivial (r < 0.1); small (0.1 ≤ r < 0.3); moderate (0.3 ≤ r < 0.5); large (0.5 ≤ r < 0.7); very large (0.7 ≤ r < 0.9); and nearly perfect (≥0.9). All the statistical procedures were executed on SPSS (version 28.0.0.0, IBM, Chicago, USA) with the significance set to p < 0.05.

## 3 Results

The descriptive statistics of skinfold measurements using Harpenden and Lipowise skinfold calipers can be found in Table 2.

**Table 2:** Descriptive statistics and within-instrument reliability measures.

	HSC	HSC	HSC	HSC	HSC	HSC	Lipowise	Lipowise	Lipowise	Lipowise	Lipowise	Lipowise
	T1	T2	T3	p	CV%	ICC	T1	T2	T3	p	CV%	ICC
Triceps (mm)	11.3 ± 5.4	11.3 ± 5.4	11.3 ± 5.4	0.698	1.29 ± 1.09	0.999 [0.999; 1.000]	11.6 ± 5.4	11.5 ± 5.4	11.5 ± 5.4	0.396	2.43 ± 2.20	0.998 [0.997; 0.999]
Subscapular (mm)	10.1 ± 3.2	10.1 ± 3.2	10.1 ± 3.2	0.954	1.31 ± 1.15	0.999 [0.998; 0.999]	10.7 ± 3.4	10.6 ± 3.3	10.5 ± 3.3	<0.001	2.07 ± 1.03	0.998 [0.996; 0.999]
Biceps (mm)	4.5 ± 2.3	4.5 ± 2.4	4.6 ± 2.4	0.073	2.89 ± 2.71	0.998 [0.996; 0.999]	4.4 ± 2.0	4.5 ± 2.0	4.5 ± 2.1	0.646	3.82 ± 3.20	0.997 [0.994; 0.998]
Iliac (mm)	11.3 ± 4.5	11.4 ± 4.6	11.4 ± 4.6	0.452	2.03 ± 1.66	0.998 [0.997; 0.999]	11.7 ± 4.5	11.8 ± 4.6	11.8 ± 4.5	0.249	2.86 ± 1.75	0.997 [0.995; 0.998]
Abdominal (mm)	14.1 ± 5.6	14.2 ± 5.7	14.3 ± 5.7	0.168	1.68 ± 1.50	0.999 [0.998; 0.999]	14.2 ± 5.9	14.1 ± 5.8	14.0 ± 5.8	0.209	1.84 ± 1.14	0.999 [0.998; 0.999]
Supraspinal (mm)	8.0 ± 3.1	8.0 ± 3.0	7.9 ± 3.0	0.040	1.49 ± 1.15	0.999 [0.998; 1.000]	8.7 ± 3.4	8.7 ± 3.6	8.7 ± 3.6	0.716	2.91 ± 1.84	0.998 [0.996; 0.999]
Thigh (mm)	15.9 ± 8.1	15.8 ± 8.1	15.9 ± 8.1	0.585	1.87 ± 3.92	0.996 [0.993; 0.998]	16.0 ± 8.2	16.1 ± 8.3	16.2 ± 8.5	0.509	2.33 ± 1.48	0.998 [0.997; 0.999]
Medial calf (mm)	8.8 ± 5.2	8.8 ± 5.1	8.8 ± 5.1	0.494	2.14 ± 2.36	1.000 [0.999; 1.000]	8.9 ± 5.3	8.8 ± 5.2	8.8 ± 5.2	0.290	2.36 ± 1.76	0.999 [0.999; 1.000]

HSC: Harpenden skinfold caliper; ICC: intraclass correlation test performed with the two-way random (absolute agreement), average measures; p: significance value for the repeated measures ANOVA.

ICC was between 0.996 and 1.000 for both instruments, which suggests excellent reliability. Additionally, the coefficient of variation ranged between 1.29 and 2.89% for different skinfold measures with the Harpenden caliper and between 1.84 and 3.82% with the Lipowise caliper.

Table 3 presents the concurrent validity inspection performed between Harpenden and Lipowise.

**Table 3:** Concurrent validity (Harpenden and Lipowise) of different skinfold values calculated as the median of three trials.

	HSC	Lipowise	%dif	p	r [95%CI] |p
Triceps (mm)	11.3 ± 5.4	11.5 ± 5.3	1.7	0.878	r = 0.991 [0.982; 0.995] |p < 0.001
Subscapular (mm)	10.1 ± 3.2	10.6 ± 3.3	5.0	0.497	r = 0.982 [0.964; 0.990] |p < 0.001
Biceps (mm)	4.5 ± 2.3	4.5 ± 2.0	1.1	0.948	r = 0.724 [0.519; 0.845] |p < 0.001
Iliac (mm)	11.3 ± 4.6	11.7 ± 4.5	3.6	0.684	r = 0.982 [0.965; 0.991] |p < 0.001
Abdominal (mm)	14.2 ± 5.6	14.1 ± 5.8	0.4	0.975	r = 0.952 [0.907; 0.974] |p < 0.001
Supraspinal (mm)	8.0 ± 3.0	8.7 ± 3.5	9.4	0.320	r = 0.884 [0.782; 0.937]; p < 0.001
Thigh (mm)	15.9 ± 8.1	16.0 ± 8.3	1.1	0.925	r = 0.988 [0.977; 0.994] |p < 0.001
Medial calf (mm)	8.8 ± 5.2	8.8 ± 5.3	0.3	0.980	r = 0.993 [0.986; 0.996] |p < 0.001
Sum of skinfolds (mm)	84.0 ± 32.0	86.0 ± 32.6	2.4	0.788	r = 0.988 [0.977; 0.994] |p < 0.001

HSC: Harpenden skinfold caliper; %dif: percentage of difference between HSC and Lipowise; r: correlation coefficient.

**Fig. 3:**
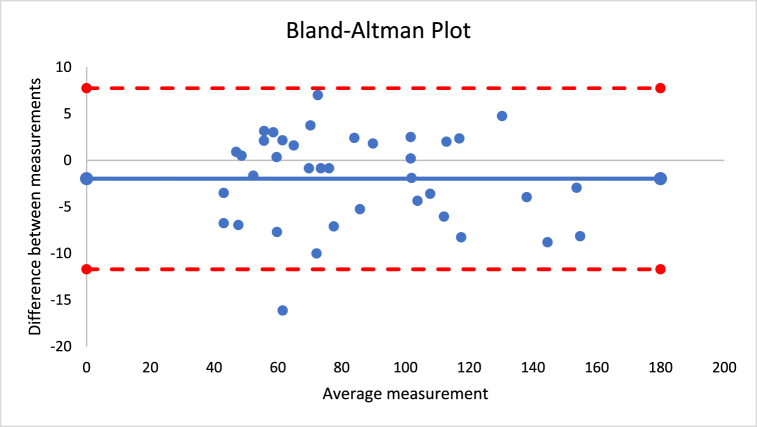
Bland-Altman plot for sum of skinfolds using Harpenden and Lipowise calipers.

The percentage of difference for the different outcomes varied between 0.3 and 5.0%. No significant differences were found between instruments considering all the outcomes (p > 0.05). The correlation coefficients were between 0.724 and 0.991, which suggests very large to nearly perfect correlations. Fig. 3 presents the Bland-Altman plot of both instruments for the sum of skinfolds outcome. The mean difference was −2, with a lower limit of −12 and an upper limit of 8, as established by a 95% confidence interval.

Table 4 presents the concurrent validity between DXA and the Harpenden and Lipowise calipers for the main outcome of all variables relate to body composition.

**Table 4:** Concurrent validity of the different variables’ outcomes calculated using dual-energy X-ray absorptiometry and the Harpenden and Lipowise caliper.

	DEXA	HSC	Lipowise	DXA vs. HSC %Dif	DXA vs. Lipowise %Dif	HSC vs. Lipowise %Dif	DXA vs. HSC p	DXA vs. Lipowise p	HSC vs. Lipowise p	DXA vs. HSC r [95%CI] |p	DXA vs. Lipowise r [95%CI] |p	HSC vs. Lipowise r [95%CI] |p
Muscle mass (kg)	28.4 ± 6.3	28.4 ± 6.4	27.1 ± 6.8	0.9	5.0	4.8	0.001	<0.001	0.809	r = 0.955 [0.913; 0.976] p < 0.001	r = 0.954 [0.910; 0.975] p < 0.001	r = 0.999 [0.998; 0.999] p < 0.001
%BF (%)	22.0 ± 7.4	17.2 ± 7.7	16.8 ± 7.7	27.9	31.0	2.4	<0.001	<0.001	0.005	r = 0.815 [0.664; 0.898] p < 0.001	r = 0.814 [0.662; 0.897] p < 0.001	r = 0.993 [0.987; 0.996] p < 0.001
FFM (kg)	50.2 ± 10.6	55.1 ± 12.5	55.4 ± 12.4	8.9	9.4	0.5	0.002	0.001	0.009	r = 0.630 [0.381; 0.787] p < 0.001	r = 0.638 [0.393; 0.792] p < 0.001	r = 0.999 [0.998; 0.999] p < 0.001

%BF: body fat percentage; FFM: fat-free mass; HSC: Harpenden skinfold caliper; DXA: Dual-energy X-ray absorptiometry.

Repeated measures revealed significant differences between muscle mass when calculated using Harpenden (p = 0.001) and Lipowise (p < 0.001) based on the Lee equation, compared to muscle mass when calculated using DXA. No significant differences were found between the Harpenden and Lipowise calipers (p = 0.809). We also observed no significant differences in repeated measures between %BF and FFM obtained from DXA and the values estimated by applying the Eston equation to the skinfold measurements (%BF_DXA_harpenden_ - <0.001; %BF_DXA_lipowise_ - <0.001; %BF_harpenden_lipowise_ – 0.005; %FFM_DXA_harpenden_ - 0.002; FFM_DXA_lipowise_ - 0.001; FFM_harpenden_lipowise_ – 0.009).

**Fig. 4:**
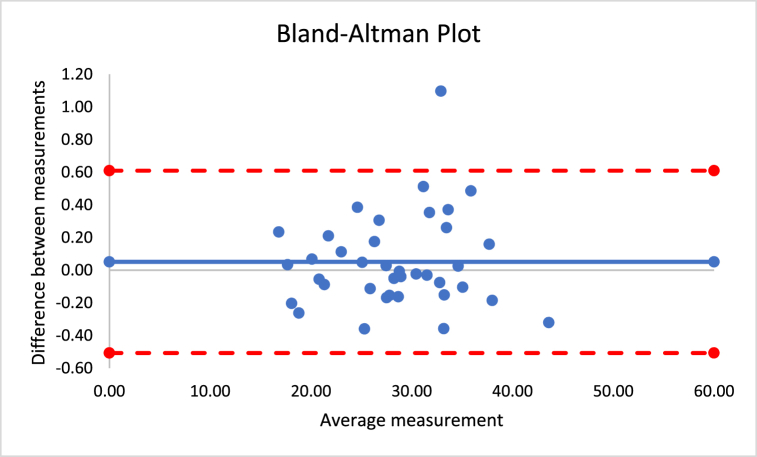
Bland-Altman plot for muscle mass using Harpenden and Lipowise calipers.

**Fig. 5:**
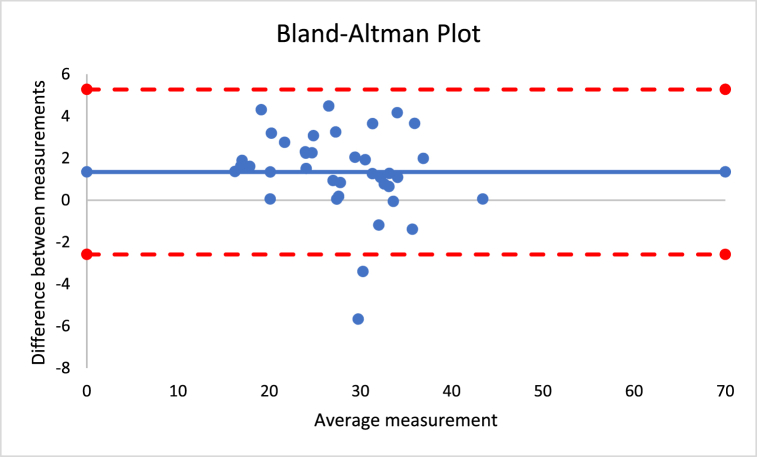
Bland-Altman plot for muscle mass using Harpenden caliper and DXA.

**Fig. 6:**
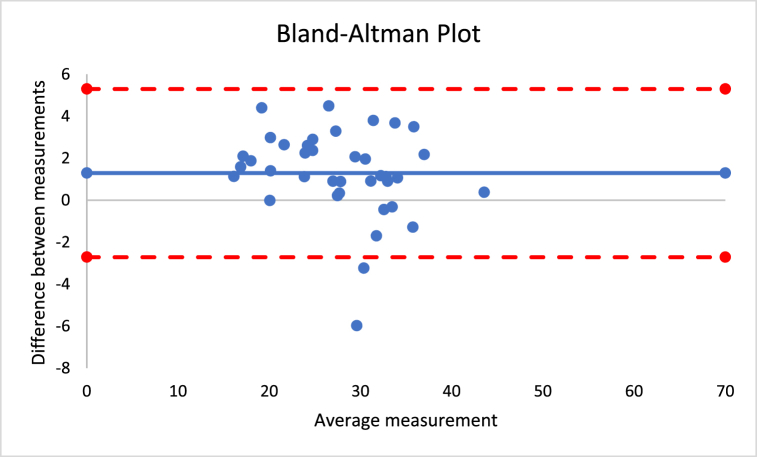
Bland-Altman plot for muscle mass using Lipowise caliper and DXA.

**Fig. 7:**
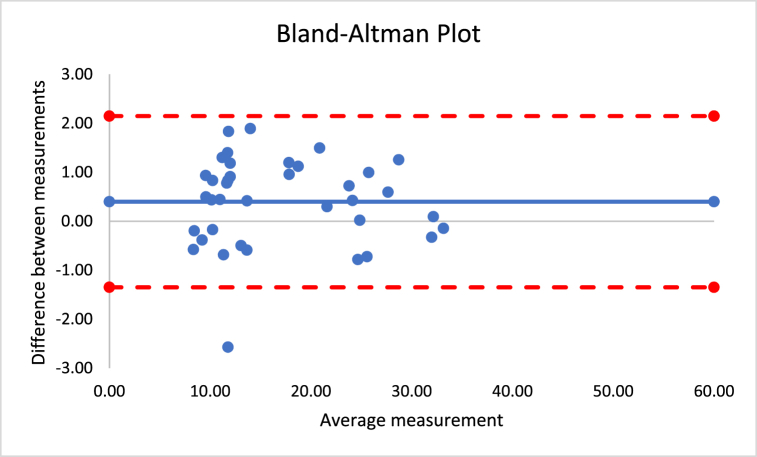
Bland-Altman plot for %BF using Harpenden and Lipowise calipers.

**Fig. 8:**
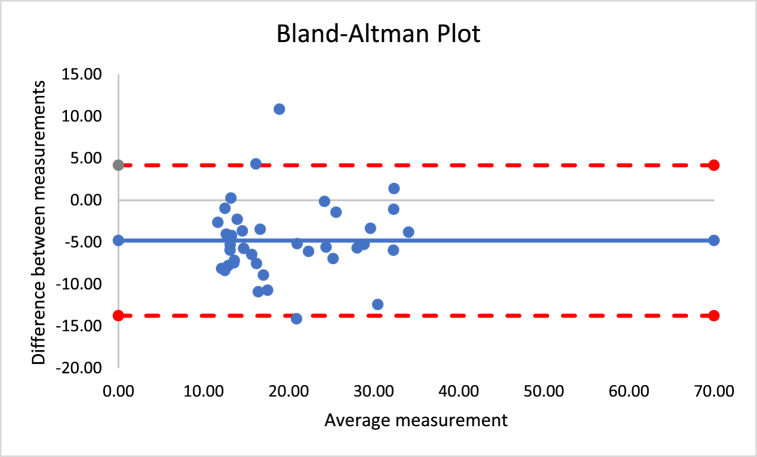
Bland-Altman plot for %BF using Harpenden calipers and DXA.

**Fig. 9:**
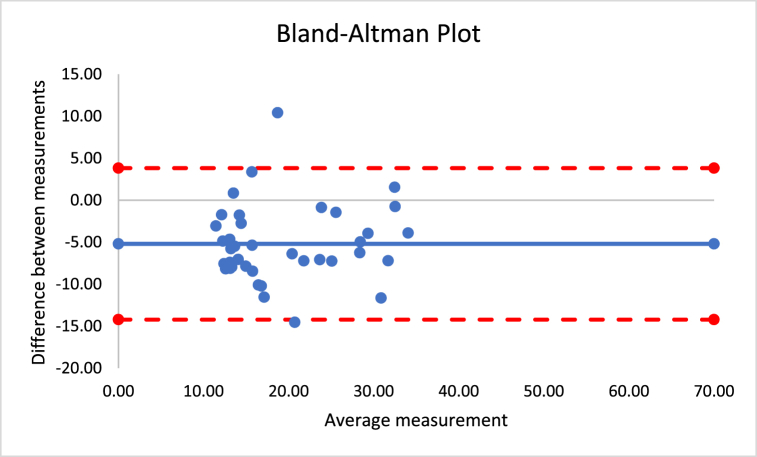
Bland-Altman plot for %BF using Lipowise calipers and DXA.

**Fig. 10:**
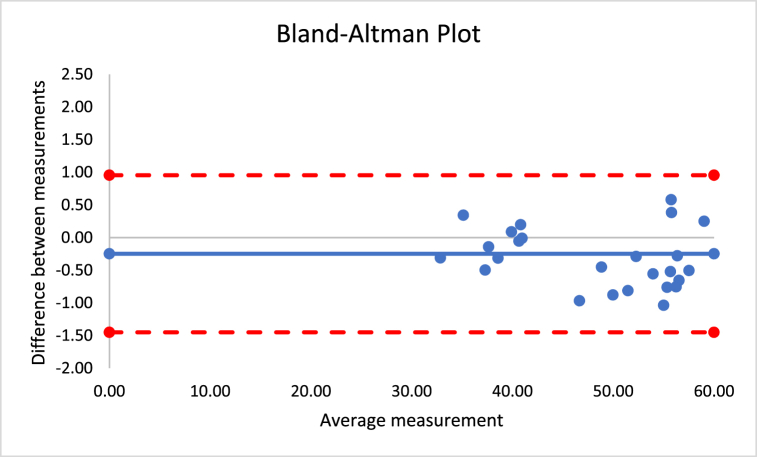
Bland-Altman plot for FFM using Harpenden and Lipowise calipers.

**Fig. 11:**
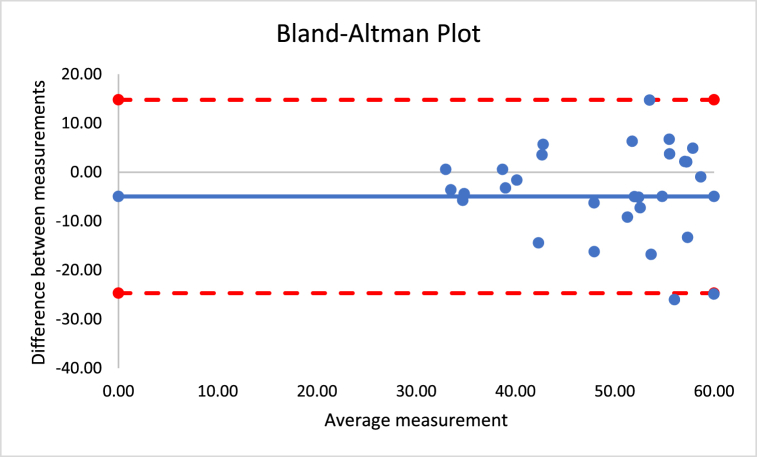
Bland-Altman plot for FFM using DXA and Harpenden calipers.

**Fig. 12:**
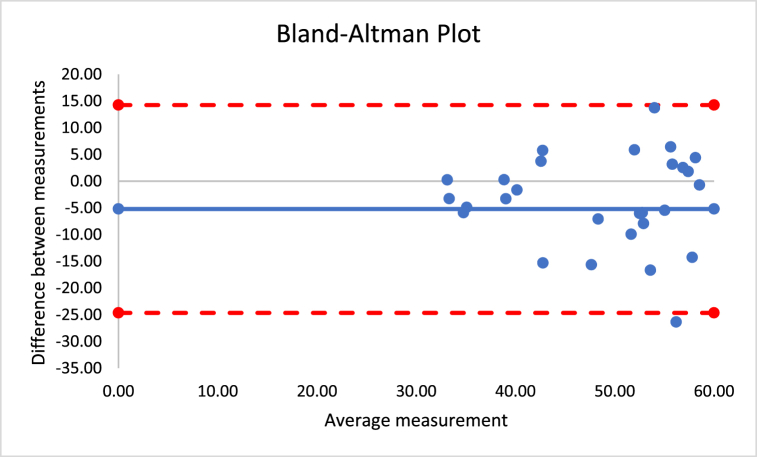
Bland-Altman plot for FFM using DXA and Lipowise calipers.

The correlations revealed that DXA muscle mass, %BF, and FFM are nearly perfectly correlated with both Harpenden (r = 0.955; r = 0.815; r = 0.630) and Lipowise (r = 0.954; r = 0.814; r = 0.638). Fig. 4, Fig. 5, Fig. 6, Fig. 7, Fig. 8, Fig. 9, Fig. 10, Fig. 11, Fig. 12 present the Bland-Altman plot of DXA and the Harpenden and Lipowise calipers for muscle mass, %BF, and FFM.

The mean difference between Harpenden and Lipowise (Fig. 4) was 0.1, with a lower limit of −0.5 and an upper limit of 0.6, as established by a 95% confidence interval.

The mean difference between Harpenden and DEXA (Fig. 5) was 1.3, with a lower limit of −2.6 and an upper limit of 5.3, as established by a 95% confidence interval.

The mean difference between Lipowise and DXA (Fig. 6) was 1.3, with a lower limit of −2.7 and an upper limit of 5.3, as established by a 95% confidence interval.

The mean difference between Harpenden and Lipowise (Fig. 7) was 0.4, with a lower limit of −1.3 and an upper limit of 2.1, as established by a 95% confidence interval.

The mean difference between Harpenden and DXA (Fig. 8) was 4.8, with a lower limit of −13.8 and an upper limit of 4.2, as established by a 95% confidence interval.

The mean difference between Lipowise and DXA (Fig. 9) was 5.2, with a lower limit of −14.2 and an upper limit of 3.8, as established by a 95% confidence interval.

The mean difference between Harpenden and Lipowise (Fig. 10) was 0.2, with a lower limit of −1.5 and an upper limit of 1.0, as established by a 95% confidence interval.

The mean difference between Harpenden and DXA (Fig. 11) was 5.0, with a lower limit of −24.7 and an upper limit of 14.8, as established by a 95% confidence interval.

The mean difference between Lipowise and DXA (Fig. 12) was 5.2, with a lower limit of −24.6 and an upper limit of 14.3, as established by a 95% confidence interval.

## 4 Discussion

This study aimed to evaluate a novel skinfold caliper's ability to assess skinfolds by evaluating its validity and reliability. Furthermore, we aimed to compare the muscle mass, %BF, and FFM assessed with DXA and estimated from the values of the skinfolds and perimeters of the participants.

The main findings were that the Lipowise skinfold caliper presents similar results with a small magnitude of difference. No statistically significant differences between the measured values were detected when compared to the Harpenden skinfold caliper for the eight assessed skinfolds, indicating a high degree of reliability with nearly perfect correlations.

Since the Harpenden skinfold caliper has been the most widely used [12,13] and is considered the gold standard model [10], these results correspond with those observed in three other studies that compared the two skinfold calipers: Harpenden with Lipowise or its beta version—the LipoTool [17,24,25]. For instance, in a similar study on university students, Amaral et al. compared the Harpenden skinfold caliper to the Lipowise precursor system (the LipoTool). They found correlation coefficients of 0.91 (p < 0.001) in every skinfold, which, despite representing a very large correlation, were slightly lower than the ones found in our study [17]. In an elderly population, Restivo et al. used the LipoTool and found a correlation of 0.997, showing strong agreement between the Harpenden skinfold caliper and this new instrument [24]. Finally, more recently, Esparza-Ros et al. investigated a similar sample as that of the present study and found an almost perfect correlation coefficient between the Lipowise and the Harpenden skinfold calipers, showing values above 0.98 (p < 0.001) [25].

Small differences are sometimes found in the pressure exerted by the calipers [26], provided that the mechanical pressure characteristics are within the norms for these instruments namely, average pressures of 10.00 g/mm^2^ on the ascending scale and 8.25 g/mm^2^ on the descending scale [27,28]. However, no considerable differences were observed between the skinfold calipers in the present study. Considering this, previous studies comparing similar tools (though they did not intend to validate the Lipowise instrument) obtained results comparable to ours. The between-instrument correlations in these studies range from 0.96 to 0.99, and validity values range from 0.8 to 0.85 when the fat mass percentage estimates were compared with the values of the skinfolds for assessments obtained using gold standard methods, such as DXA or hydrostatic weighing [11,14,29,30].

The values of muscle mass and free-fat mass were slightly overestimated, while %BF was underestimated. Nevertheless, the narrow limits of agreement from the Bland-Altman plot confirm the results of the measurements of different skinfolds and %BF calculations when estimated using equations derived from anthropometry relative to muscle mass, FFM, and %BF derived from DXA. This finding supports the Lipowise digital system's accuracy. The current results also confirm the findings of previous studies reporting similar results comprising different samples and showing strong associations between body fat percentage assessed with DXA and the sum of the skinfolds [[31], [32], [33], [34]]. Furthermore, the correlations observed in our study are similar to those reported in previous studies that used fat mass as the criterion variable, with previous studies reporting correlation values superimposed on ours [17,24,25].

Furthermore, the data showed the same pattern regarding muscle mass. Both calipers showed good validity concerning muscle mass, %BF, and FFM assessed with DXA and the same variables estimated from skinfolds and girth values. A comparison of results assessed by the two methods yielded a very large correlation between DXA and both calipers for muscle mass and %BF(DXA-MuscleMassHarp – 0.955; DXA-MuscleMassLipo – 0.954; DXA-%BFharp – 0.815; DXA-%BFlipo – 0.814), a large correlation for FFM (DXA-FFMharp – 0.630; DXA-FFMlipo – 0.638), and a very large correlation between the values found with the calipers for all variables (MuscleMassHarp-MuscleMassLipo – 0.999; %BFharp-%BFlipo – 0.993; FFMharp-FFMlipo – 0.993). Nevertheless, to the best of our knowledge, this is the first study to use muscle mass as a validity criterion to demonstrate that the Harpenden and the Lipowise calipers are valid tools for assessing muscle mass among healthy young adults considering Lee et al.‘s [22] formula.

This study has some limitations. First, since participants were recruited on a convenience basis, some caution should be taken when extrapolating the current findings to other samples. Moreover, although the measurer who recorded the data was an ISAK level-2 accredited kinanthropometrist with a low TEM and significant experience, this could be another source of error. Nevertheless, since there was only one measurer and no alternative to the protocol was used in the investigation, the measurement method likelydid not affect the validity of the different skinfold calipers analyzed.

## 5 Conclusions

The present study provided evidence that the Lipowise skinfold caliper, a novel piece of equipment used to assess skinfolds, is an accurate instrument and represents an innovation in skinfold thickness and body composition evaluation based on anthropometric measurements.

Furthermore, the Lipowise skinfold caliper can be an alternative tool for technicians who need to assess body fat or muscle mass in a precise, valid, and time-efficient way since it increases the simplicity of recording the data and enables subjectivity to be removed from the interval of time after applying the caliper and registering the skinfold value.

It should be noted, however, that some caution should be taken when using these skinfold calipers interchangeably when evaluating skinfolds. It is advisable to perform measurements with the same model from the same brand of skinfold caliper when performing follow-up assessments.

## Author contribution statement

César Leão: Filipe Manuel Clemente: Bruno Silva: Conceived and designed the experiments; Analyzed and interpreted the data; Contributed reagents, materials, analysis tools or data; Wrote the paper.

Georgian Badicu: Performed the experiments.

Miguel Camões: José Maria Cancela: Joel Pereira: Performed the experiments; Analyzed and interpreted the data; Contributed reagents, materials, analysis tools or data; Wrote the paper.

## Data availability statement

Data will be made available on request.

## Declaration of competing interest

The authors declare that they have no known competing financial interests or personal relationships that could have appeared to influence the work reported in this paper
